# Vulnerability extraction and prediction method based on improved information gain algorithm

**DOI:** 10.1371/journal.pone.0309809

**Published:** 2024-09-10

**Authors:** Peng Yang, Xiaofeng Wang

**Affiliations:** School of Computer Science and Engineering, North Minzu University, Yinchuan, China; Jinan University, CHINA

## Abstract

More and more attention has been paid to computer security, and its vulnerabilities urgently need more sensitive solutions. Due to the incomplete data of most vulnerability libraries, it is difficult to obtain pre-permission and post-permission of vulnerabilities, and construct vulnerability exploitation chains, so it cannot to respond to vulnerabilities in time. Therefore, a vulnerability extraction and prediction method based on improved information gain algorithm is proposed. Considering the accuracy and response speed of deep neural network, deep neural network is adopted as the basic framework. The Dropout method effectively reduces overfitting in the case of incomplete data, thus improving the ability to extract and predict vulnerabilities. These experiments confirmed that the excellent F1 and Recall of the improved method reached 0.972 and 0.968, respectively. Compared to the function fingerprints vulnerability detection method and K-nearest neighbor algorithm, the convergence is better. Its response time is 0.12 seconds, which is excellent. To ensure the reliability and validity of the proposed method in the face of missing data, the reliability and validity of Mask test are verified. The false negative rate was 0.3% and the false positive rate was 0.6%. The prediction accuracy of this method for existing permissions reached 97.9%, and it can adapt to the development of permissions more actively, so as to deal with practical challenges. In this way, companies can detect and discover vulnerabilities earlier. In security repair, this method can effectively improve the repair speed and reduce the response time. The prediction accuracy of post-existence permission reaches 96.8%, indicating that this method can significantly improve the speed and efficiency of vulnerability response, and strengthen the understanding and construction of vulnerability exploitation chain. The prediction of the posterior permission can reduce the attack surface of the vulnerability, thus reducing the risk of breach, speeding up the detection of the vulnerability, and ensuring the timely implementation of security measures. This model can be applied to public network security and application security scenarios in the field of computer security, as well as personal computer security and enterprise cloud server security. In addition, the model can also be used to analyze attack paths and security gaps after security accidents. However, the prediction of post-permissions is susceptible to dynamic environments and relies heavily on the updated guidance of security policy rules. This method can improve the accuracy of vulnerability extraction and prediction, quickly identify and respond to security vulnerabilities, shorten the window period of vulnerability exploitation, effectively reduce security risks, and improve the overall network security defense capability. Through the application of this model, the occurrence frequency of security vulnerability time is reduced effectively, and the repair time of vulnerability is shortened.

## 1. Introduction

The development and expansion of information technology has continuously promoted the innovation and change of science and technology [1]. However, its widespread use has also brought various potential security threats [2]. Computer vulnerabilities refer to errors or vulnerabilities in software or hardware in a computer that can be exploited by hackers or malicious elements to invade systems, steal data, or disrupt services [3]. These vulnerabilities may lead to serious consequences, including data leakage, property damage, service interruption, and even pose a threat to personal safety [4]. Up to now, a lot of artificial intelligence and machine learning technologies have been applied in the field of information security, which can help the system realize automatic response to security problems more effectively and reduce the degree of manual intervention [5]. At the same time, the proposal of Extended Detection and Response (XDR) provides more perfect and in-depth threat detection and response functions for computer information security [6,7].

In 2021, the discovery of the Log4Shell (CVE-2021-44228) vulnerability widely affected the Java logging library Log4j, which allows remote code execution and can be exploited by attackers to execute malicious code without authorization, due to the widespread use of Log4j in enterprise and open source projects. The vulnerability affected almost every industry in the world, including key sectors such as government, finance, telecommunications, and education. In early 2021, several related vulnerabilities codenamed "ProxyLogon" were discovered in Microsoft Exchange Server, allowing an attacker to remotely access a Microsoft Exchange server, execute code and steal data, while the attacker could cut off corporate communications. It has a great impact on the business operation of enterprises. The Pulse Connect Secure VPN vulnerability (CVE-2021-22893), discovered in 2021 in the Pulse Connect Secure VPN product, allows unauthorized users to execute arbitrary code, An attacker can exploit a VPN vulnerability as an initial foothold, and the attacker can launch broader network attacks such as internal gateway penetration and horizontal movement. Serious vulnerability problems can lead to the disclosure of sensitive information, or cause system and network services to be interrupted. Leakage of sensitive information can lead to company leaks, which may further lead to serious business losses. The interruption of system and network services will affect normal work and learning, interfere with normal business operations, and affect the quality of life.

With the continuous improvement of data privacy regulations, privacy enhancement technology has been proposed, so that data can be encrypted without exposing the content [8]. Due to the complexity of computer and network communication, vulnerabilities are almost inevitable. Many vulnerabilities involve software coding errors, design defects, configuration issues, and hardware vulnerabilities [9]. Therefore, vulnerability protection has become an important link in ensuring system and data security. It involves various security measures and best practices, including vulnerability scanning, vulnerability patching, strong password policies, access control, firewalls, intrusion detection systems, etc. [10]. Continuous vulnerability management and security awareness training are also crucial for reducing system risks. There is often a correlation between vulnerabilities, and then multiple vulnerabilities can be concatenated, resulting in an orderly combination of vulnerabilities. The vulnerability detection method can help evaluate the potential impact of different vulnerabilities and determine the priority of repair work. Vulnerability extraction and prediction focuses on discovering security problems that may be exploited by attackers by analyzing system software or hardware defects, and further evaluating how, when and potential impact of these vulnerabilities may be exploited. Therefore, it provides forward-looking guidance for the deployment of security protection measures. Vulnerability extraction and prediction are part of vulnerability detection.

In the attack response, the vulnerability correlation can be used to analyze the correlation and dependence between vulnerabilities, so as to build a comprehensive defense. On this basis, the vulnerabilities in the system can be classified and prioritized, so that the vulnerabilities that may be jointly exploited in the attack can be repaired first. At the same time, the correlation analysis can predict the potential attack path, so as to strengthen the corresponding protection and reduce the impact of security problems. By using the vulnerability association technology, the vulnerabilities in the network system can be determined more quickly and the potential intrusion path of attackers can be predicted. Enables security teams to prioritize defense strategies based on vulnerability and attack continuity, such as prioritizing patching those vulnerabilities that play a critical role in the attack chain, thereby improving response speed in attack response. In cybersecurity research, building a vulnerability exploitation chain is a complex process that involves identifying and correlating multiple vulnerabilities in a system to form a path that an attacker could exploit. The main difficulty is that vulnerabilities may exist at multiple levels such as software, hardware and protocol, and each type of vulnerability has different characteristics and utilization methods, which increases the difficulty of extracting and associating the characteristics of vulnerabilities. The network environment is dynamic, and new software versions, system configurations, and user behaviors may affect the way and effect of vulnerability exploitation. It is a challenge to build a vulnerability exploitation chain that can adapt to this dynamic change.

By studying the vulnerability correlation, the system can quickly take appropriate measures when detecting the threat signal related to a major vulnerability, such as automatically isolating the affected network segment or deploying targeted security patches, so as to achieve effective and rapid response in the early stage of the attack. In vulnerability detection, the application of artificial intelligence and deep learning has greatly improved the accuracy and efficiency of detection, providing new opportunities for the reform of traditional vulnerability detection methods [11]. By studying this type of vulnerability chain, effective protection can be formed against multiple vulnerabilities. The study of vulnerability correlation usually involves the premise of the attack on the vulnerability and the environment after the attack, which are referred to as the pre-permission and post-permission of the vulnerability [12]. Based on the currently constructed vulnerability library, due to the lack of some vulnerability data [13], it is often difficult to mine pre-permission and post-permission for vulnerabilities, which leads to difficulties in exploiting vulnerability correlation chains. Therefore, a vulnerability mining and prediction method based on improved Information Gain (IG) is proposed. By strengthening the model’s utilization of vulnerability features, research can achieve accurate prediction of pre-permission and post-permission for vulnerabilities. This can better utilize the vulnerability correlation chain, which is conducive to the development of computer information security.

The study is divided into four parts. The first part is a summary of the current situation in the field of computer security and vulnerability security. The second part is the construction and implementation of the proposed method, which integrates Dropout strategies to enhance the ability of deep learning models to process information gain. Specifically, vulnerability data is preprocessed to screen out features with large amount of information, and then an improved information gain indicator is used to evaluate the correlation between features and vulnerabilities. By integrating Dropout, the model randomly ignores partial connections during training, thereby improving its ability to resist over-fitting and enhancing generalization performance on unknown data. In addition, this method can also build a vulnerability exploitation chain by analyzing the correlation between vulnerabilities. The third part is the validation of the proposed method, using NVD data set and introducing existing models, the performance of the proposed method is quantitatively tested. The fourth part is the summary and prospect of the research.

This study proposes a vulnerability extraction and prediction method based on an improved information gain algorithm. The method takes deep neural networks as the basic framework and introduces Dropout technology. The advantage of this technique is that it can reduce the overfitting phenomenon during model training and enhance the generalization ability of the model. Potential application areas include public network security, application security scenarios, personal computer security protection, and enterprise cloud server security. The contribution to the progress of domain knowledge and technology development lies in providing an effective solution to the problem of vulnerability feature extraction and prediction under incomplete data, which is expected to promote the development of technology and knowledge in the field of network security.

## 2. Related works

The development of the network has continuously increased the utilization rate of computers. People are increasingly concerned about the security of computers and data. Computer security refers to a series of measures and practices that protect computers and networks from un-authorized access, malicious software attacks, data breaches, service interruptions, and other threats. It involves hardware security, software security, network security, data security, and user authentication. Based on the current use of computers, Envelope JSA discusses the issue of personal privacy security based on data from the perspective of computer network, and emphasizes the importance of computer network security technology. The practical results show that the development of network security provides theoretical support [14]. Based on the field of mobile communication technology, Wu T H et al. studied the computer data security in the mobile industry, explored the correlation between relationship quality and information security, emphasized the soundness of computer audit, and prospected the future development direction of computer security [15]. Shandilya S K et al. developed a network security testing platform using attack parameters that effectively assessed network defense strategies. The successful experiments validated the platform’s capability in bolstering computer security [16]. Paul S et al. proposed a communication encryption method based on cryptography, which combined quantum communication digital signature and key construction, in the face of the severe situation of network security. And they evaluated the method using multiple different security levels. These experiments confirmed the confidentiality and authentication attributes of this method [17]. Baum C et al. introduced a forgetting linear function evaluation protocol aimed at enhancing network communication security, which demonstrated superior active security and efficiency on standard hardware [18]. Haq M A et al. proposed two Deep Neural Network models (DNNBot1 and DNNBot2) aiming at robot attacks in the Internet of Things, and the research proved that they can detect and classify attacks effectively and quickly, with excellent accuracy and efficiency [19]. Haq M A et al. proposed a deep learning-based intrusion detection method (PCCNN) for DoS and DDoS attacks in the Internet of Things system, and the research proved that it can effectively improve the detection accuracy, reaching 99.34% and 99.13% respectively [20]. For enterprise internal threat detection, Haq M A et al proposed two deep learning hybrid LSTM models (GLoVe LSTM and Word2vec LSTM), which proved to be effective in improving detection accuracy and superior to machine learning models such as XGBoost, AdaBoost, RF, KNN and LR [21]. Kavin Kumar K et al. proposed to adopt transfer learning and localization perception convolutional neural network architecture for classification and localization of large-scale fish images, and the research proved that this method effectively improved the recognition accuracy and localization accuracy [22]. Aiming at large-scale fish image recognition and localization, Ahmad U et al. proposed a CNN architecture combining transfer learning and localization perception, and the research showed that this scheme significantly improved the classification and localization performance [23]. Jawaharlalnehru A et al. proposed an image detection technology based on improved YOLO algorithm to address the accuracy challenges encountered in UAV image detection. The experimental results proved that the method had excellent accuracy and improved the detection speed [24]. Arunnehru J et al. proposed the use of machine vision to solve the problem of human motion recognition, and proposed a method that combined spatio-temporal motion features with differential intensity distance group model. Experimental results proved that this method effectively improved the accuracy rate of human motion recognition [25].

Vulnerability security is an important branch of computer security, focusing on identifying, analyzing, and patching vulnerabilities in computers and software. Munonye K et al. proposed a potential vulnerability detection method based on OAuth data to address vulnerabilities in web applications. These experiments confirmed that this method achieved a performance accuracy of over 90%, and its vulnerability identification matching rate was as high as 54% [26]. Qasem A et al. classified and evaluated vulnerability detection methods in existing embedded systems in the face of security vulnerabilities in IoT devices. And they further conducted quantitative and qualitative research on these methods. Finally, they analyzed the challenges faced by IoT security vulnerabilities and provided suggestions for future research directions in this field [27]. Paknezhad M et al. analyzed the reasons for the vulnerability of deep learning models to adversarial attacks and proposed a defensive framework based on semi-supervised learning. These experiments confirmed that the framework improved the defense performance of the original model, and the model still maintained high performance [28]. Gu H et al. crafted a web software vulnerability detection method to counter SQL injection attacks on cloud servers, notable for its ability to identify, analyze, and provide immediate feedback on security threats, thereby strengthening computer information security [29]. Lachkov P et al. conducted vulnerability simulation and attack simulation on the web platform aiming at common network security threats and attacks in computer systems, and proposed a penetration attack test based on the real situation, the main purpose of which is to determine the vulnerability of the network and the impact of the attack. The experimental results show that this method can provide a comprehensive test of network security, thus enhancing network security and playing a strong role in promoting the field of computer security [30].

To sum up, in the face of the current severe network security situation, the security of computer systems and data is constantly emphasized. In order to achieve more accurate identification and detection of security vulnerabilities, considering the rapid development of computer network vulnerabilities, and in order to realize real-time response and identification of vulnerabilities, a vulnerability extraction and prediction method based on improved information gain algorithm is proposed. Firstly, the collected vulnerability data is thoroughly preprocessed, including data cleaning, normalization and coding, to ensure that the features in the data set can reflect the normal and abnormal behavior of the system. A deep neural network is then used to assess statistical correlations between data features and known vulnerabilities in order to more accurately identify potential vulnerabilities. Dropout technology is further used to enhance the generalization ability of the model and avoid over-fitting phenomena. By utilizing the correlation of vulnerabilities, a chain of vulnerability exploitation can be effectively constructed, enabling rapid and effective response measures to be taken during vulnerability attacks. Therefore, a vulnerability extraction and prediction method based on the improved IG is proposed. By using Dropout to improve DNN’s IG ability and enhance its vulnerability extraction ability, vulnerability extraction and prediction can be achieved.

## 3. Using improved IG for vulnerability extraction and prediction

To effectively defend against vulnerabilities, it is necessary to fully utilize the correlation of vulnerabilities and construct an effective vulnerability exploitation chain. In the study, DNN is used as the basic algorithm for vulnerability extraction prediction model. Considering the shortcomings of its IG, Dropout is used to improve it and strengthen its feature extraction for vulnerabilities. And combined with the vulnerability permission generation method used in practice, the effective extraction and prediction of vulnerabilities are achieved.

### 3.1. Selection and construction of IG

**Fig 1:**
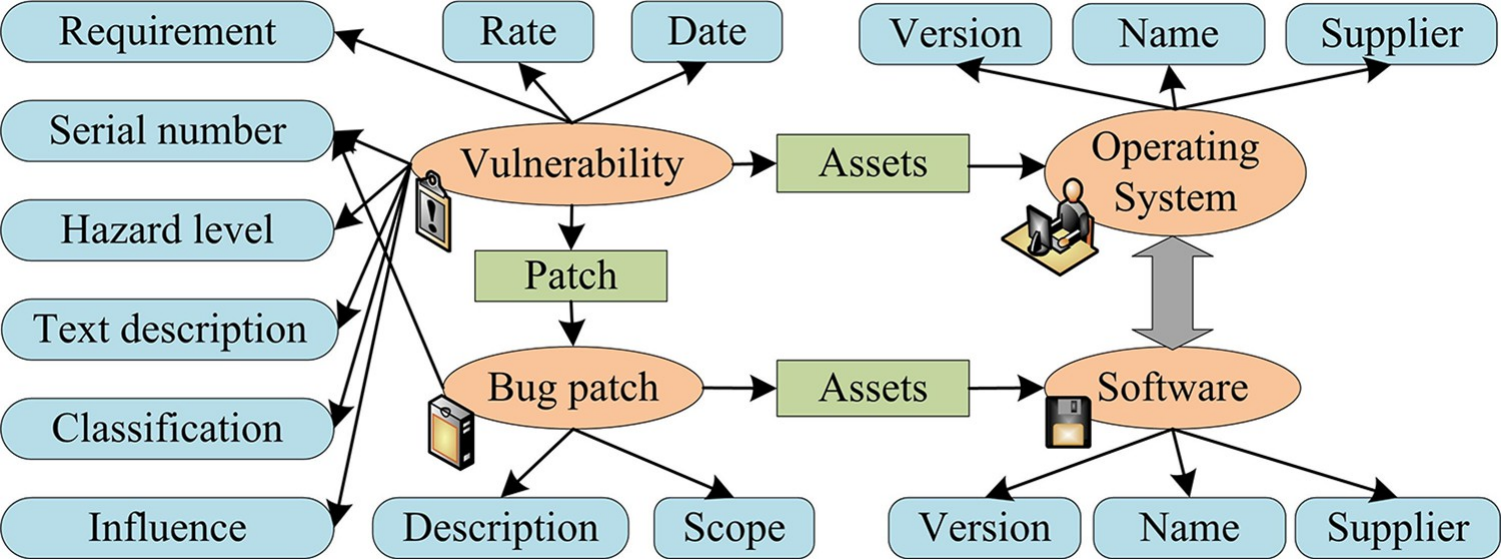
Information contained in the usual vulnerability library.

In order to maximize the correlation between vulnerabilities and build a utilization chain between vulnerabilities, it is necessary to study the prerequisites for the occurrence of vulnerabilities and the environment after their occurrence, that is, to generate pre-permission and post-permission for vulnerabilities [31]. Vulnerability libraries are commonly used public data bases in computer security to collect and organize a large amount of vulnerability information. They contain various types of vulnerabilities, such as software, hardware, protocols, etc. Vulnerability libraries are usually maintained by security researchers, vendor organizations, or relevant communities, and are regularly updated. The importance of vulnerability libraries lies in providing a centralized source of information for security researchers, practitioners, and users to understand existing and newly discovered vulnerabilities. By using vulnerability libraries, users can track the latest vulnerability dynamics in a timely manner and take corresponding security measures as needed to protect the security of the system and data. Fig 1 shows the information contained in common vulnerability libraries.

**Fig 2:**
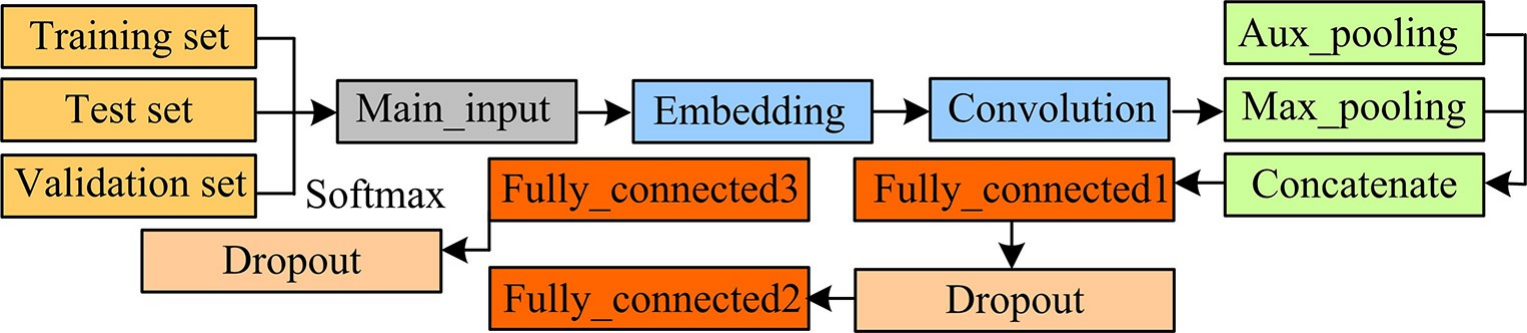
Neural network extraction vulnerability data flow chart.

Usually, in vulnerability libraries, pre-permission and post-permission are missing due to the lost data. Pre-permission refers to the conditions that attackers need to meet before exploiting a specific vulnerability. These conditions may include access permissions to the target system, specific environmental settings, user permissions, etc. If these pre-permissions are missing from the vulnerability library, users may not fully understand the exploitability and impact of specific vulnerabilities. Post-permission refers to the permissions or actions that an attacker may obtain or perform after successfully exploiting a vulnerability. For example, a vulnerability can enable an attacker to gain administrator privileges or access other sensitive data. If the detailed information on these post-permissions is not provided in the vulnerability library, users may not be able to accurately evaluate the threat level and response measures. The lack of data makes it difficult to build a vulnerability exploitation chain, so DNN is used to generate pre-permission and post-permission for vulnerabilities. Feed-forward Neural Network (FFNN), also known as Multi-layer Perceptron (MLP), mainly consists of three layers [32]. In FFNN, data are passed forward from the inputting layer to the outputting layer without feedback connections. Each neuron node receives input from the previous layer and passes it on to the next layer. Fig 2 shows the flow chart of using neural network to extract vulnerability data in the study.

In FFNN, Formula (1) is the net input of neurons.


$$z^{l}=W^{l}a^{l-1}+b^{l}$$ (1)

In Formula (1), *z*^*l*^ represents the net input of the *l*-th layer neurons. *a*^*l*−1^ represents the input of the (*l*−1)-th layer neuron. *W*^*l*^ represents the weight matrix from layers *l*−1 to *l*. *b*^*l*^ represents the offset vector from layers *l*−1 to *l*. Formula (2) is the input for neurons.


$$a^{l}=f_{l}(z^{l})$$ (2)

In Formula (2), *a*^*l*^ represents the input of the *l*-th layer neuron. *f*_*l*_(∙) represents the activation function of the (*l*−1)-th layer neurons. Combining Formulas (1) and (2), the net input of neurons can be further represented by Formula (3).


$$z^{l}=W^{l}f_{l-1}(z^{l-1})+b^{l}$$ (3)

Combining Formulas (1) and (2), the neuron input can be further represented by Formula (4).


$$a^{l}=f_{l}(W^{l}a^{l-1}+b^{l})$$ (4)

The composite function *θ*(*x*,*W*,*b*) is used to represent FFNN. The neuron input of the input layer is *a*^0^ = x. The input can be obtained through iteration in Formula (5).


$$x=a^{l}\to z^{1}\to a^{1}\to z^{2}\to \cdots \to a^{L-1}\to z^{L}\to a^{L}=\theta (x,W,b)$$ (5)

**Fig 3:**
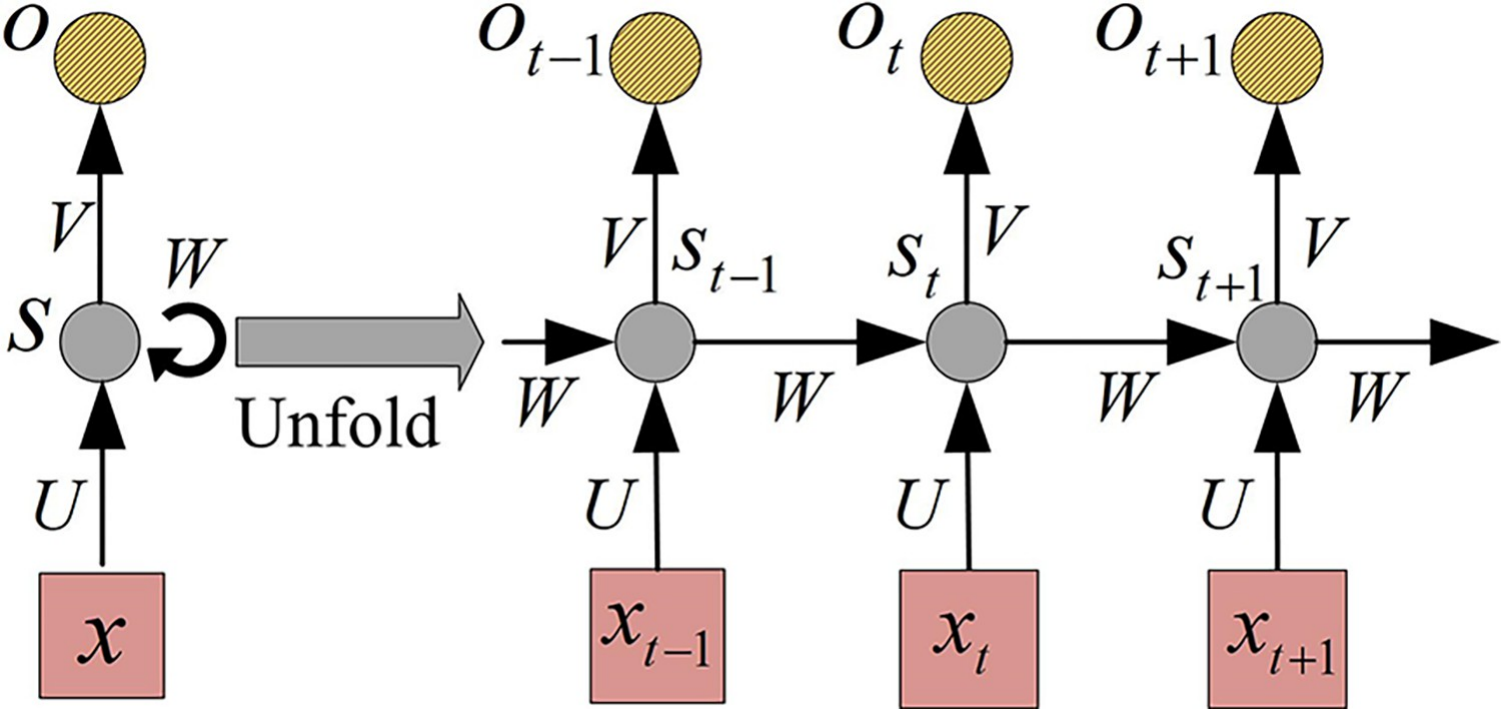
Schematic diagram of recurrent neural network forward propagation framework.

*x* represents a word vector. After iteration, the output *a*^*L*^ of the *L*-th layer neurons can be obtained. For sequence data, Recurrent Neural Network (RNN) is usually used for processing. Compared to FFNN, RNN can receive sequences of any length, which is limited to output length. In the training of RNN, there are two types of operations: forward propagation and back-propagation. In forward propagation, RNN processes input sequences step by step in time and calculates and predicts them based on connection weights [33]. At each time step, RNN receives the input of the current time step and the hidden state of the previous time step, and then generates the hidden state and output of the current time step through the operation of the activation function and weight matrix. This process continues until the last time step of the input sequence. Fig 3 shows forward propagation.

At *t* moment, Formula (6) represents the output of hidden neurons.


$$h_{t}=Ux_{t}+Ws_{t-1}$$ (6)

In Formula (6), *h*_*t*_ represents the output of hidden layer neurons at time *t*. *U* represents the weight matrix of the hidden layer-hidden layer. *x*_*t*_ represents the temporal data input at time *t*. *W* represents the weight matrix of the input layer-hidden layer. *s*_*t*−1_ represents the state of the hidden unit at time *t*−1. Formula (7) represents the state of hidden unit.


$$s_{t}=f(h_{t})$$ (7)

In Formula (7), *f*(∙) represents the nonlinear activation function. Formula (8) is the final output value.


$$y_{t}=g(Vs_{t})$$ (8)

In Formula (8), *g*(∙) represents the nonlinear activation function. *V* represents the weight matrix of the hidden layer output layer. The output of the hidden layer is usually associated with the current input and the previous output, which gives RNN the ability to process sequence data. In forward propagation, the weight matrix is usually updated through back-propagation. At time *t*, each output will generate an error. Formula (9) represents the total error.


$$E=\sum_{i=0}^{t}e_{i}$$ (9)

In RNN, the output of each moment needs to consider the network state of the previous moment, so the temporality is added in back-propagation. When *t* = 3, the chain derivative method in Formula (10) can be used.


$$\frac{\partial E_{3}}{\partial W}=\frac{\partial E_{3}}{\partial y_{3}}\frac{\partial y_{3}}{\partial s_{3}}\frac{\partial s_{3}}{\partial W}$$ (10)

In Formula (10), *s*_3_ is not only associated with the weight matrix of the input layer-hidden layer, but also with *s*_2_, so it can be further represented by Formula (11).


$$\frac{\partial s_{3}}{\partial W}=\frac{\partial s_{3}}{\partial s_{3}}\frac{\partial s_{3}^{+}}{\partial W}+\frac{\partial s_{3}}{\partial s_{2}}\frac{\partial s_{2}}{\partial W}$$ (11)

In Formula (11), $\frac{\partial s_{3}^{+}}{\partial W}$ represents the direct derivative of *W* after removing the influence of previous moments. Similarly, the expansions of $\frac{\partial s_{2}}{\partial W}$ and $\frac{\partial s_{1}}{\partial W}$ can be obtained and incorporated into Formula (11) to obtain Formula (12).


$$\frac{\partial E_{3}}{\partial W}=\sum_{j=0}^{3}\frac{\partial E_{3}}{\partial y_{3}}\frac{\partial y_{3}}{\partial s_{3}}\frac{\partial s_{3}}{\partial s_{j}}\frac{\partial s_{j}^{+}}{\partial W}$$ (12)

Due to the equal importance of the weight matrix between the hidden layer and the input layer, $\frac{\partial E_{3}}{\partial U}$ can be represented by Formula (13).


$$\frac{\partial E_{3}}{\partial U}=\sum_{j=0}^{3}\frac{\partial E_{3}}{\partial y_{3}}\frac{\partial y_{3}}{\partial s_{3}}\frac{\partial s_{3}}{\partial s_{j}}\frac{\partial s_{j}^{+}}{\partial U}$$ (13)

Because the weight matrix of the hidden layer output layer is only related to the output value, $\frac{\partial E_{3}}{\partial U}$ can be further obtained in Formula (14).


$$\frac{\partial E_{3}}{\partial V}=\frac{\partial E_{3}}{\partial y_{3}}\frac{\partial y_{3}}{\partial V}$$ (14)

By utilizing the sequence data processing capability of RNN, the data in the vulnerability library are processed to achieve the extraction and prediction of vulnerabilities. The model is trained using labeled vulnerability data. By optimizing the weights and biases of this model through forward and backward propagation, it learns the features and patterns of vulnerabilities. The trained RNN model is used in this experiment to extract vulnerabilities in un-labeled data. The un-labeled data are input into the model, and the predictive ability of the model is used to determine whether there were vulnerabilities. Based on the probability distribution or category labels output by the model, data containing vulnerabilities are identified and extracted.

### 3.2. Construction of a vulnerability extraction and prediction system based on improved IG

**Fig 4:**
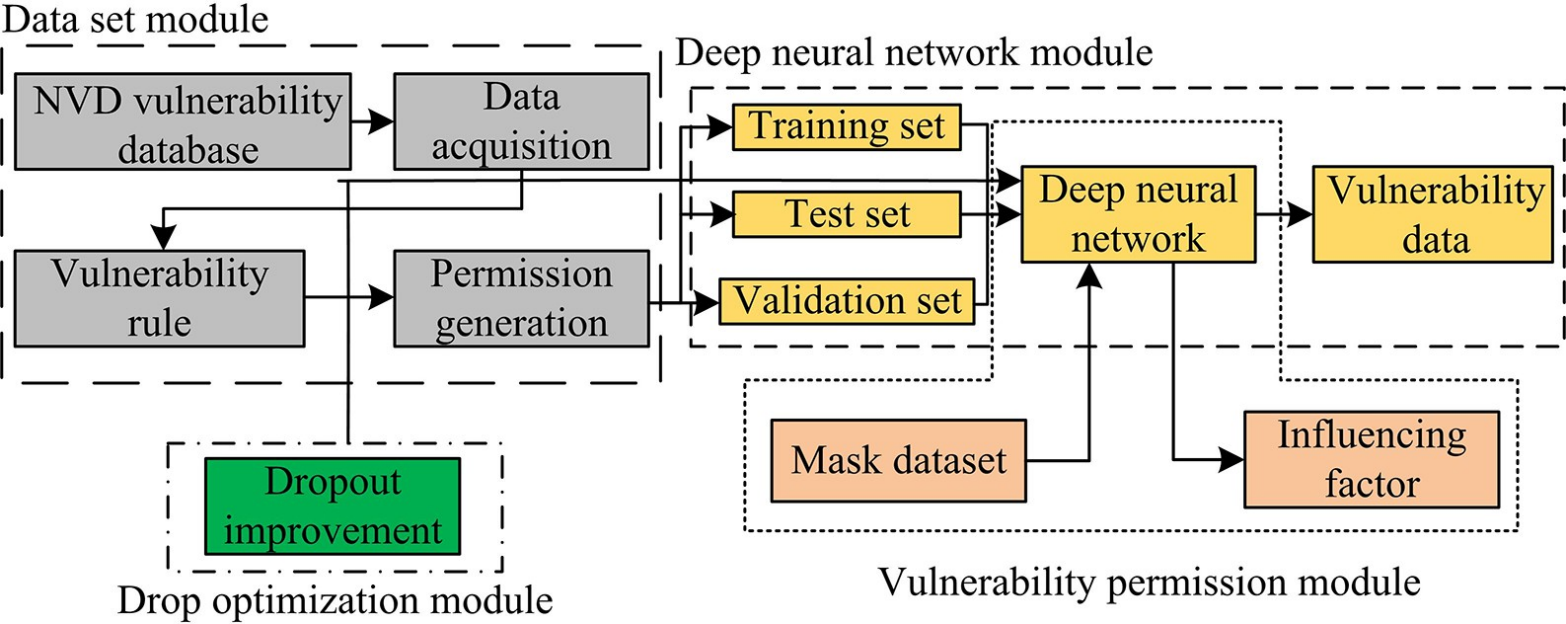
Schematic diagram of vulnerability extraction and prediction.

A DNN-based vulnerability permission generation model is proposed based on current vulnerability permission generation methods. The model mainly includes the following four parts. The first is the data set, whose main function is to design and collect vulnerability data sets. The second part is DNN, with the main functions of constructing and training DNN. The third part is vulnerability permissions, which mainly analyzes the factors that generate vulnerabilities and their impact. Finally, there is optimization, with the main function of improving the IG capability. Fig 4 shows the vulnerability extraction and prediction system constructed in the study.

**Fig 5:**
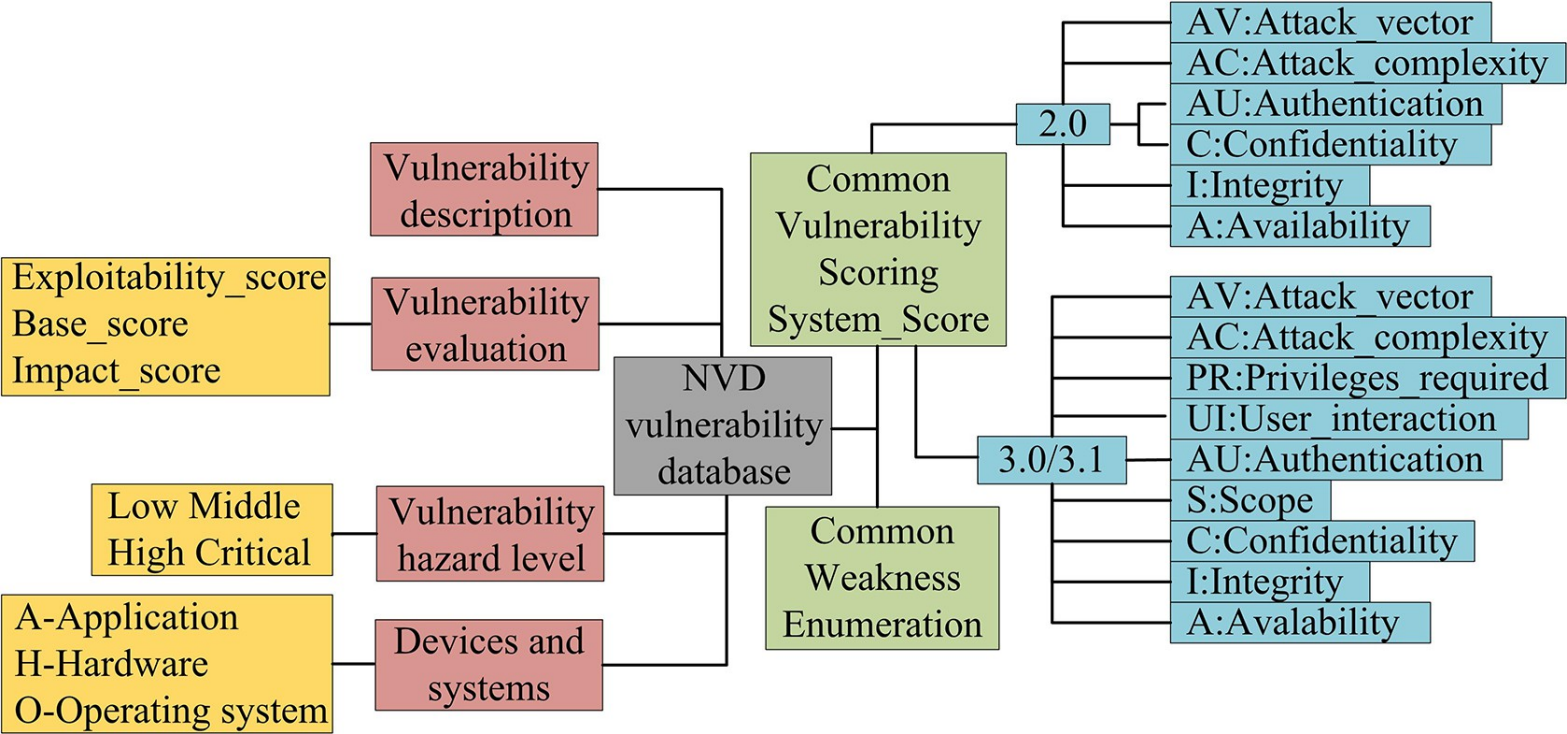
National vulnerability database information composition diagram.

Because the data sets used in research are usually searched using matching rules, the data planes obtained through this method are relatively limited and may be accompanied by information and data gaps. Therefore, the selection of vulnerability data set has considerable importance. If vulnerabilities with larger weight values can be obtained in permission generation, it can effectively improve the efficiency of vulnerability extraction and prediction. The database is used for further research. National Vulnerability Database (NVD) is a widely used and publicly available vulnerability information repository maintained by National Institute of Standards and Technology (NIST). NVD aims to collect, organize, and publish vulnerability information worldwide to help users understand, evaluate, and respond to potential security risks in software and systems. NVD is an important source of information for security practitioners, security researchers, and software developers. It can help users track and understand the latest vulnerability information, evaluate its potential threats to existing systems and software, and take timely security measures to protect the security of systems and data. Fig 5 shows the main information of NVD.

**Fig 6:**
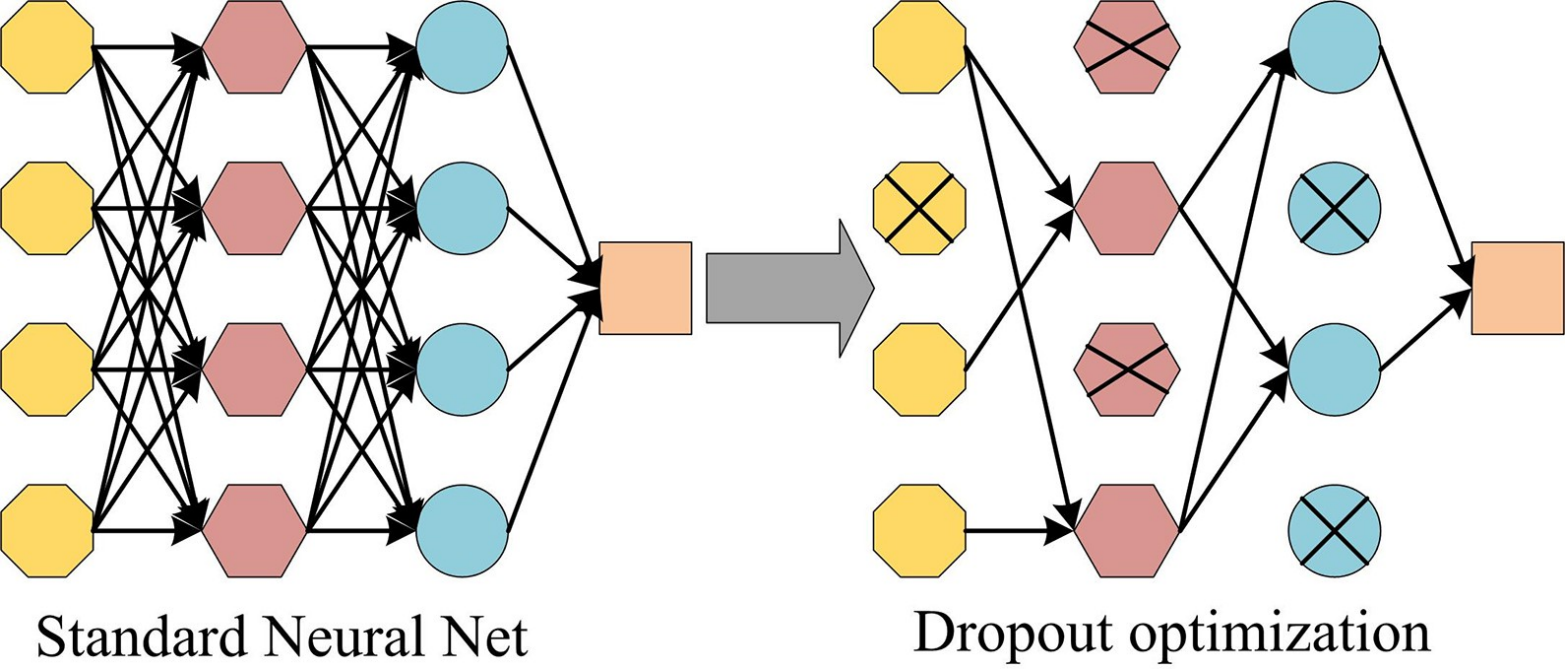
Schematic diagram of the Dropout optimization.

By adopting NVD, a series of problems caused by missing data in other vulnerability data sets can be effectively solved. Compared to other vulnerability data sets, NVD, as an officially maintained database, typically has better data integrity, reliability, and breadth, and contains a large amount of vulnerability information. As a global vulnerability database, NVD not only organizes and publishes its own collected vulnerability information, but also shares and exchanges data with other vulnerability databases. This data collaboration and supplementation enable NVD to have high data integrity. In addition, NVD also associates vulnerability information with CVE numbers, making it easier to uniformly identify and locate vulnerabilities. This can effectively enhance the vulnerability permission generation ability of the model, thereby enhancing its ability to extract and predict vulnerabilities. Due to over-fitting causing an increase in the error of DNN, the study chose to use Dropout to improve it. Fig 6 is a schematic diagram of the principle of Dropout [34].

Dropout is a commonly used regularization technique used to reduce over-fitting in neural networks. It is achieved by randomly setting the output of some neurons to zero during the training process. The main advantages of Dropout are that it can reduce over-fitting in neural networks and improve the generalization ability. By randomly discarding some neurons, the network is not dependent on any specific neuron, thereby reducing the complex mutual adaptation relationships between neurons. This forces the network to learn more robust and generalized features. In addition, Dropout can also improve computational efficiency during the training process, as only a portion of neurons in each training sample are updated in each batch, reducing computational costs. A naive Bayesian weighting method is used to confirm the importance of words in text data. In category *c*, Formula (15) is the weight of word *w*.


$$r_{c}^{w}=\frac{\left(n_{c}^{w}+1)/\|n_{c}\right\|}{\left(n_{c^{'}}^{w}+1)/\|n_{c^{'}}\right\|}$$ (15)

In Formula (15), $n_{c}^{w}$ represents the occurrences of *w* in *c*. $n_{c^{'}}^{w}$ represents the occurrences of *w* in other categories. ‖*n*_*c*_‖ represents the number of all words in category *c*. ‖*n*_*c*'_‖ represents the sum of all words in other categories. However, due to the possibility of identifying low-frequency words as important words in this method, the method has been improved, and the improved importance score is represented by Formula (16).


$$r_{c}^{w}=\frac{\left(n_{c}^{w}+1)/\|n_{c}\right\|}{\left(n_{c^{'}}^{w}+1)/\|n_{c^{'}}\right\|}\times \log_{\beta }n_{c}^{w}$$ (16)

In Formula (16), $\log_{\beta }n_{c}^{w}$ represents the frequency factor and *χ* represents the hyper-parameter. For Dropout, its hyper-parameter usually involves Dropout rate, which is the probability of randomly dropping neurons during training. If the setting is too high, the model may lose too much information, resulting in under-fitting and insufficient learning ability; Conversely, if the setting is too low, it may not be effective enough to prevent over-fitting. The choice of hyper-parameters usually needs to be determined experimentally. This involves testing the model’s performance on the validation set for different hyper-parameter values and selecting those hyper-parameter values that give the model the best performance on the validation set. The hyper-parameter in the study was set to 0.3. To optimize it using Dropout, it is necessary to calculate the Dropout probability of the importance score, as shown in Formula (17).


$$p(r)=\frac{w^{r}+1}{w^{r}-1}$$ (17)

**Fig 7:**
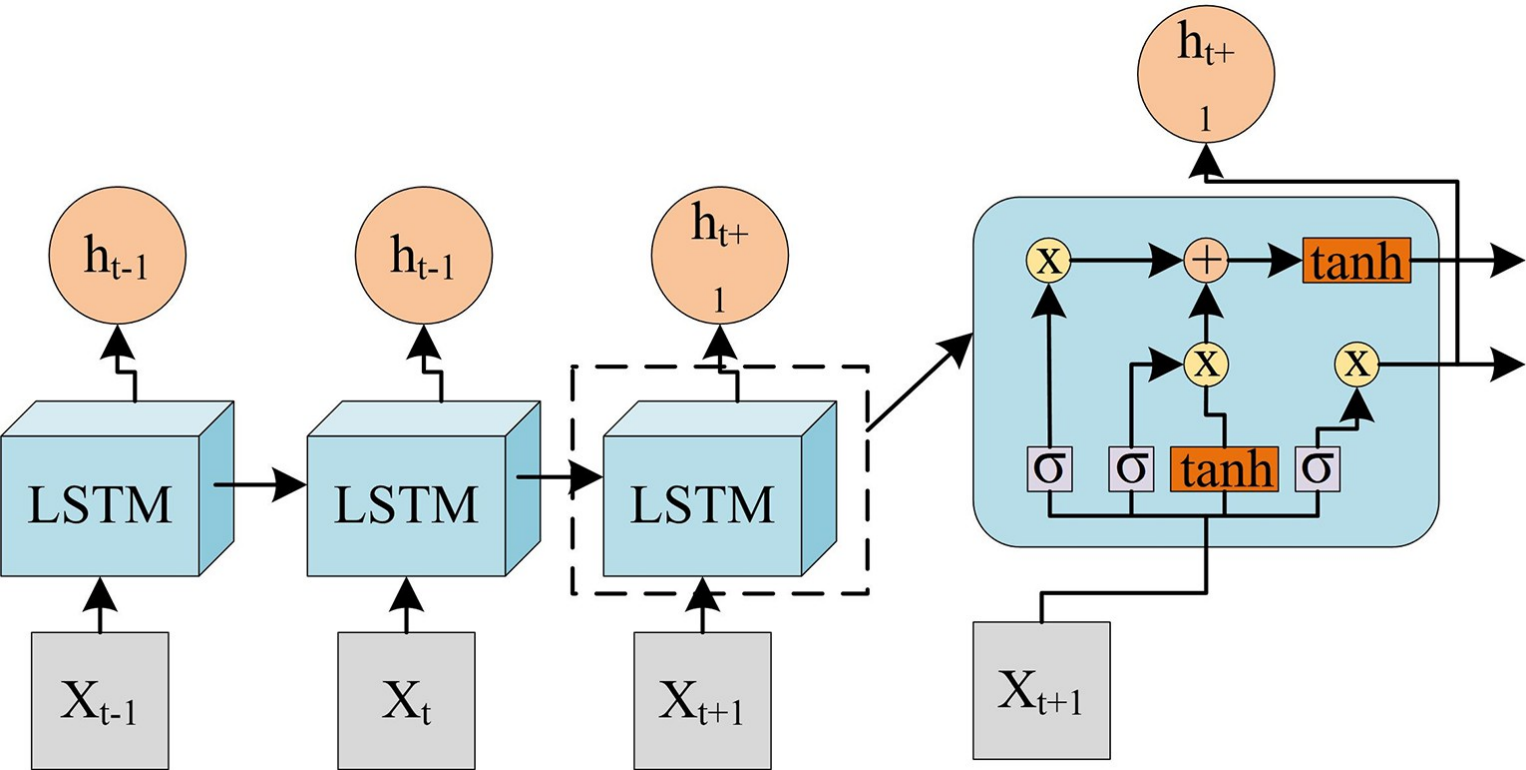
Schematic diagram of the LSTM structure.

When calculating the probability of Dropout, if the final probability is 0, *w* should be retained every time the vulnerability statement is trained. The input in the study is first entered into the BiLSTM model for further processing. The LSTM model structure adopted in the study is shown in Fig 7.

**Fig 8:**
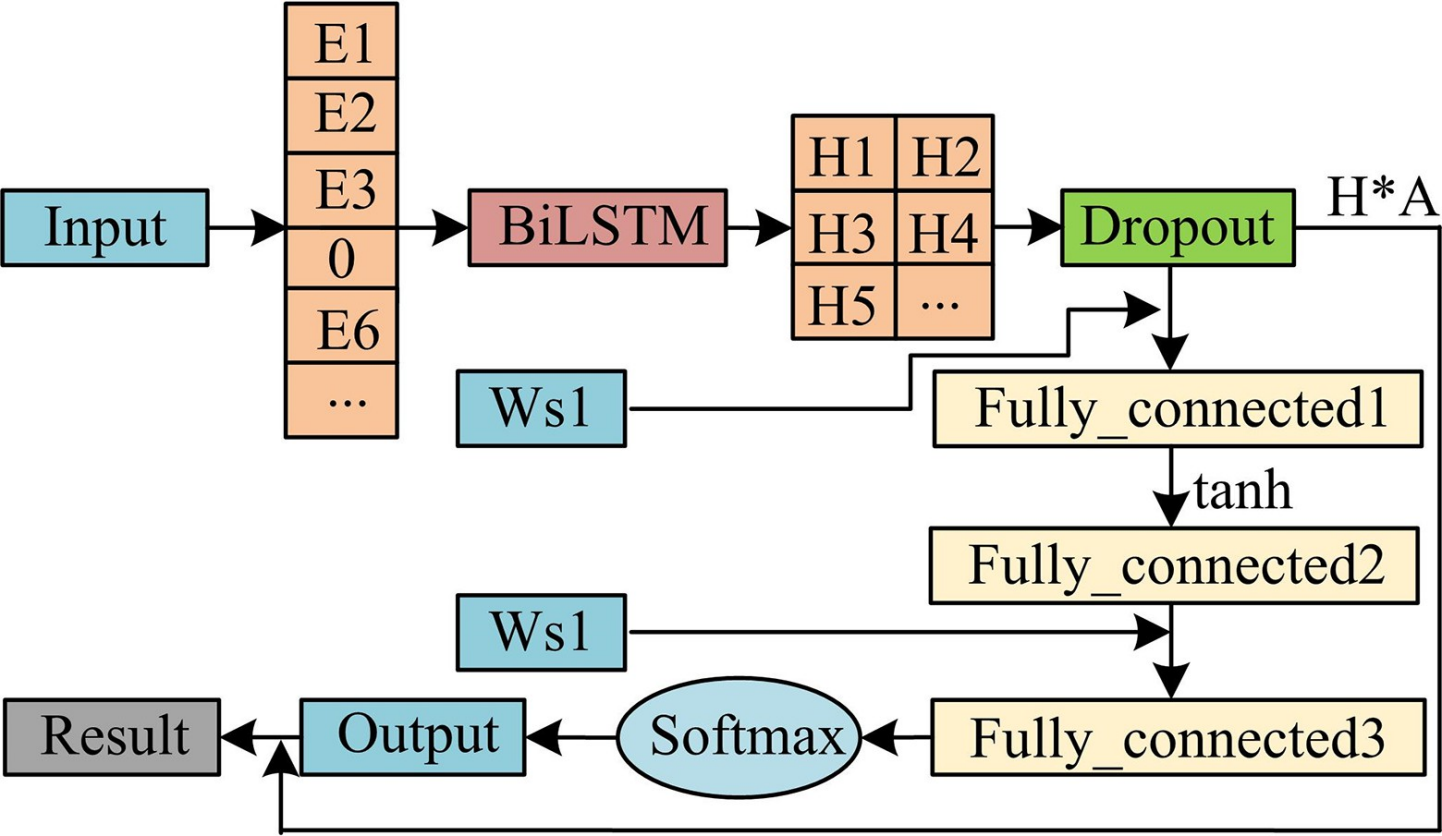
Dropout optimization model framework structure diagram.

To further enhance the model’s ability to extract vulnerability features, a self-attention mechanism is further introduced. Fig 8 shows the improved model.

**Fig 9:**
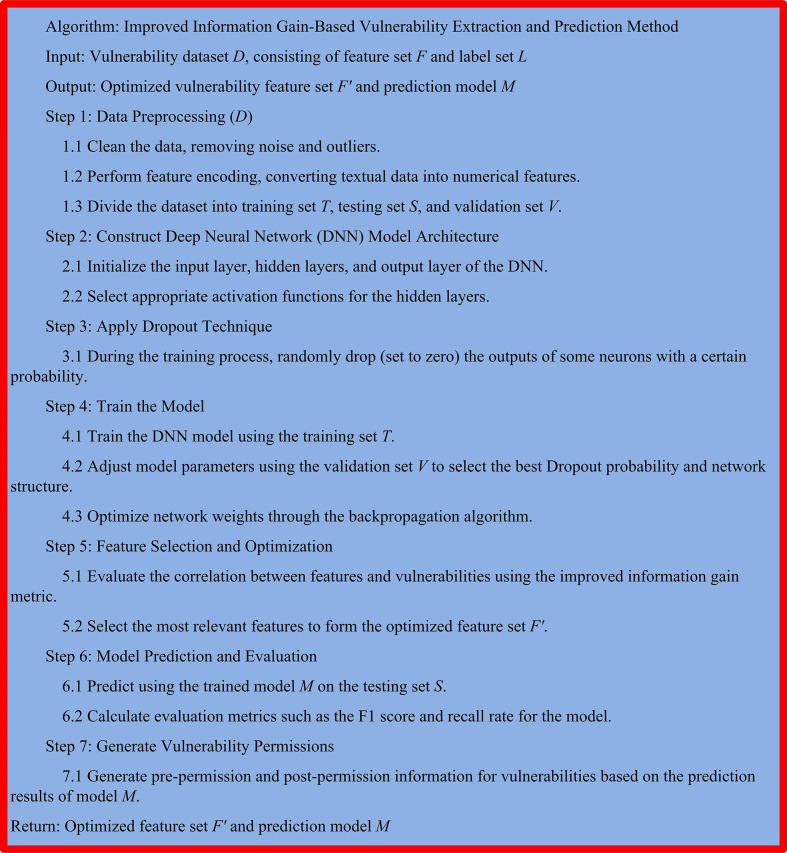
Pseudo-code of our method.

By introducing a self-attention mechanism, the model can allocate different attention weights based on the inter-relationships between different elements within the vulnerability sequence data. This can enable the model to more effectively extract the correlation between vulnerabilities. And the self-attention mechanism can significantly improve the modeling ability of traditional sequence models for long-distance dependencies. In RNN or CNN, information can only be transmitted through a limited length context window. The self-attention mechanism can consider all positions of the input sequence and learn the correlation weights of each position, thereby achieving more comprehensive extraction and prediction of vulnerabilities. The pseudo-code for the research method is shown in Fig 9.

## 4. Performance testing of vulnerability extraction and prediction methods based on improved IG

**Fig 10:**
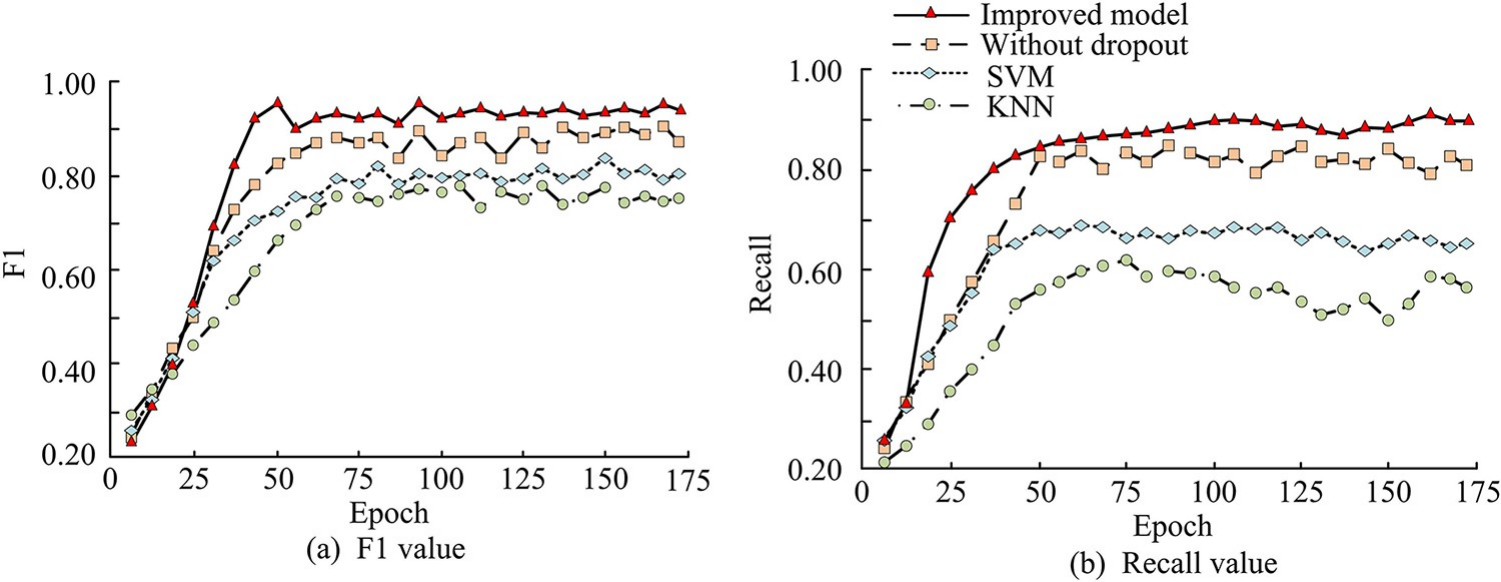
Basic performance test result of four models.

To verify the effectiveness of the proposed method, NVD was used, with 60% set as training set, 20% set as testing set, and 20% set as validation set (https://nvd.nist.gov/). First download the latest vulnerability data and extract relevant characteristics such as CVE detailed description, CVSS score, affected software/hardware version, etc. The data is then preprocessed, including text cleaning (removing punctuation, stopping word processing), encoding conversion, label classification (vulnerability/non-vulnerability), etc. Finally, it is randomly divided to ensure the uniform distribution of vulnerability types in each set. Support vector machine (SVM), K-nearest Neighbor (KNN), and the study model without Dropout improvement were introduced for comparison. First, the basic performance of the model is tested, and the test results are shown in Fig 10. K-fold cross-validation is used in this test, and K selection is 5. For each fold of cross-validation, the model is trained and the model performance is evaluated on the retained validation set, recording its recall rate and F1 score. The improved model had the highest F1, with a maximum value of 0.972, an improvement of 0.110 compared to the model without improvement, and an improvement of 0.192 and 0.264 compared to FF and KNN, respectively. Its Recall was the best, with a maximum value of 0.968, which was 0.107–0.284 higher than the other three models.

Then, a training set was used to train all models to the best state, and their convergence performance was tested. The test data were the mean value and standard deviation of the model convergence performance. Firstly, the convergence performance value of the model in each experiment was obtained by testing, and then the average value was obtained by dividing by the total number of experiments. For the standard deviation, the difference between the performance value of each experiment and the mean is first squared, then the squared squared values are summed, and this synthesis is then divided by the number of experiments minus one to obtain an unbiased estimate of the statistic. Finally, take the square root of the variance to obtain the standard deviation. In order to minimize the impact of errors, five experiments were conducted on all models, and their performance measurements were recorded after each test. In the test results in Table 1, the improved method had the best convergence mean and convergence standard deviation, proving its excellent training performance. It had lower training costs and time, and could achieve the optimal state of the model more quickly.

**Table 1:** Comparison of convergence performance of four models.

Number of experiments		Improved model	Without Dropout	SVM	DKK
1	Mean	**0.000005417**	0.000067752	0.00054127	0.00941941
	Standard	**0.000003512**	0.000055454	0.00065892	0.00042153
2	Mean	**0.000006821**	0.000009515	0.00048135	0.00584331
	Standard	**0.000006534**	0.000084362	0.00068134	0.00064513
3	Mean	**0.000004281**	0.000069557	0.00069538	0.00694821
	Standard	**0.000003369**	0.000018354	0.00067158	0.00693547
4	Mean	**0.000005327**	0.000049214	0.00036954	0.00092548
	Standard	**0.000006583**	0.000006354	0.00025417	0.00693354
5	Mean	**0.000005482**	0.000065231	0.00065814	0.00026489
	Standard	**0.000006892**	0.000009531	0.00095248	0.00158264

**Fig 11:**
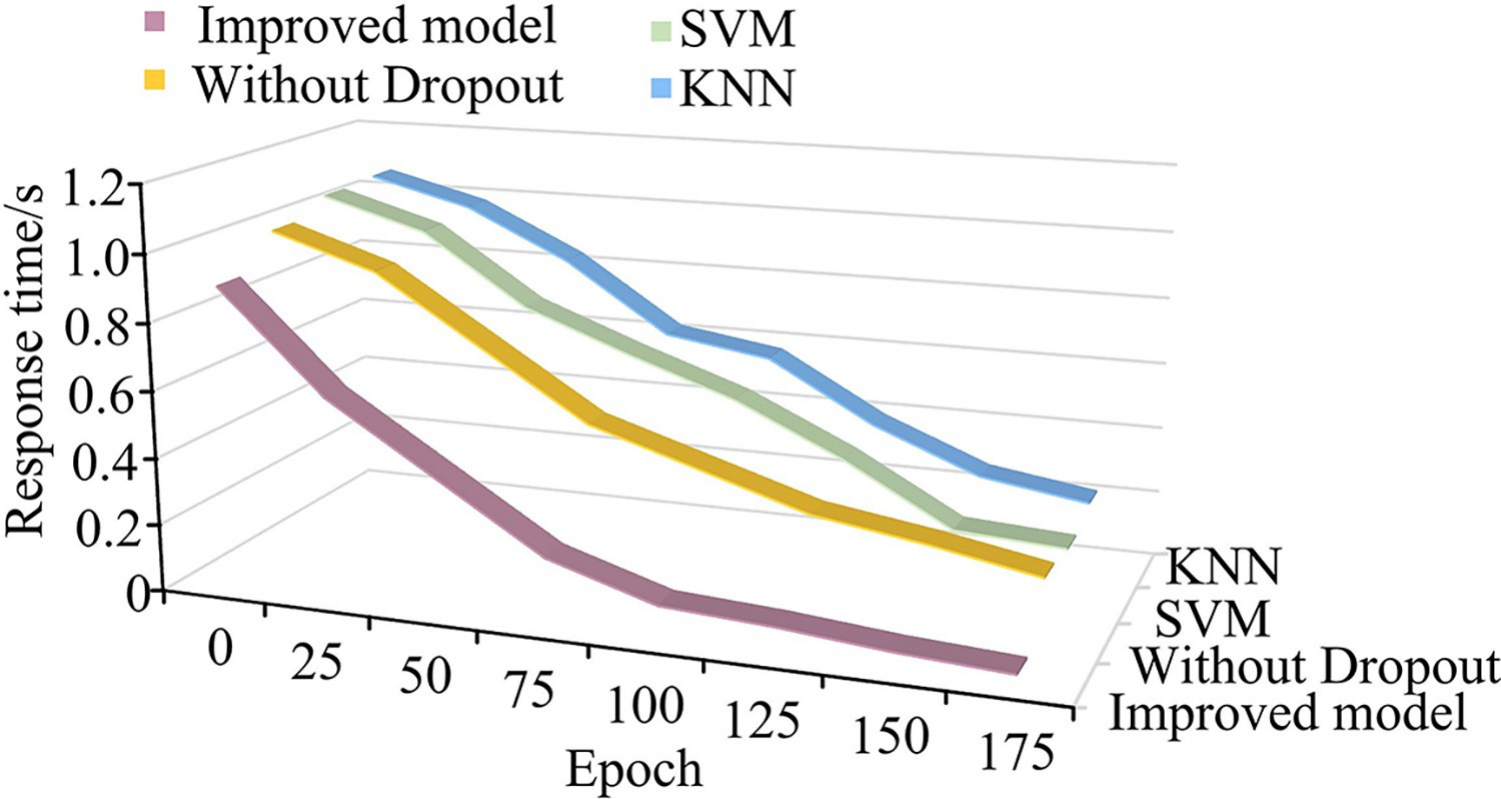
Comparison of response time of four models.

The response times of the four models were tested to assess how quickly they extracted vulnerability information, first preheating the model and then using a time timing tool to record the full time it took the model to receive input to output results. The test results are shown in Fig 11. The improved method ultimately achieved a response time of 0.12s, a reduction of 0.11s compared to the un-improved method, and a reduction of 0.16s and 0.21s compared to FF and KNN, respectively. Therefore, the improved method could output data more quickly in practical applications and achieve rapid response to vulnerabilities.

**Fig 12:**
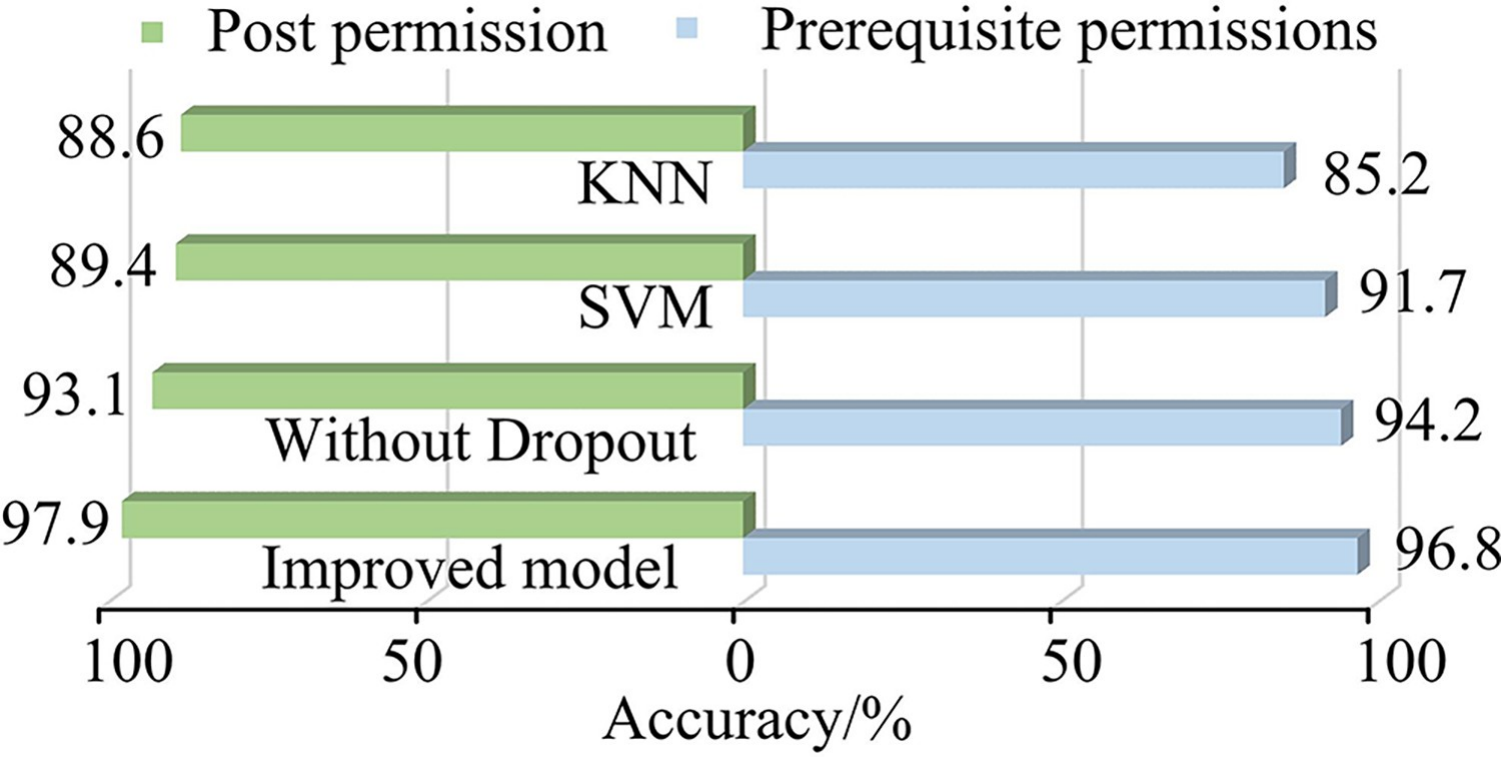
Comparison of the predictive ability of four models of pre-permission and post-permission.

It was very important to determine the pre-permission and post-permission of vulnerabilities in vulnerability analysis and prediction. Because these permissions determine whether attackers could exploit vulnerabilities to obtain un-authorized access or perform malicious operations. Therefore, the ability to generate pre-permissions and post-permissions of the four models on the overall data set was tested, specific test cases were created, including expected pre-conditions and post-conditions, and the accuracy of model generation was recorded by running these test cases through the model, with accuracy as the testing indicator in Fig 12. The improved model achieved a pre-permission generation accuracy of 97.9%, which was 4.8% higher than the un-modified model, 8.5% higher than FF and 9.3% higher than KNN, respectively. The generation accuracy of its post-permission reached 96.8%, which was 2.6% higher than the un-improved model, 5.1% higher than FF and 11.6% higher than KNN, respectively.

**Fig 13:**
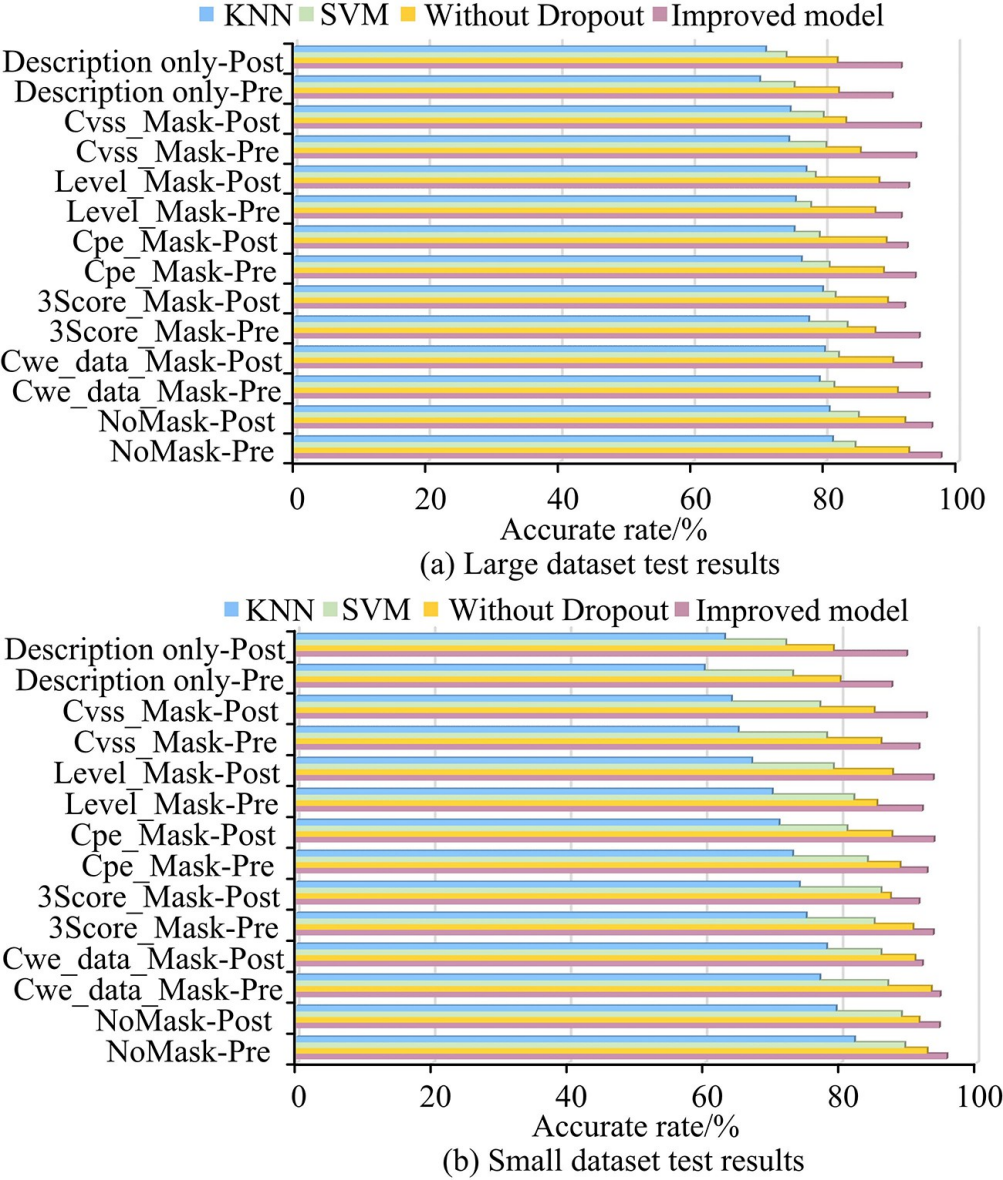
Comparison of mask test of four models.

To delve deeper into the model’s capability to handle vulnerability permission generation, the dataset utilized for this purpose was divided into two subsets. The division was not equal; rather, a 90% of the data formed the larger subset, while the remaining 10% constituted the smaller subset. This split was intended to assess the model’s performance across datasets of different sizes. For the training process, the larger data subset was further subdivided, adhering to a fixed proportion of 60% for training, 20% for validation, and 20% for testing. This distribution ensures that the model is not only trained on a substantial portion of the data but also validated and tested on significant portions to ascertain its generalization and performance. The investigation then focused on the influence of various vulnerability-related information on the model’s ability to accurately identify and predict vulnerabilities. This was conducted by applying different ‘masks’ to the data, which is a method of selectively hiding certain information from the model during training and testing to understand the importance of that information. The testing metrics of interest were the model’s accuracy in identifying vulnerabilities before and after permissions were applied. These permissions could be seen as access rights or authorizations concerning vulnerabilities. Fig 13 shows the test results. "Post" indicated that the test was post-permission, while "Pre" indicated that the test was pre-permission. "Description only" indicated that only the vulnerability descriptions in the data were retained. Cvss_Mask represented removing Common Vulnerability Scoring System data from input data. Level_Mask represented the removal of hazard level data from input data. Cpe_Mask represented the universal platform enumeration information retrieved from the data. 3Score_Mask represented removing vulnerability rating data from input data. Cwe_Mask represented removing CWE data from input data. No_Mask represented the input of complete information. From Fig 13, the testing performance within a large data set was significantly better than a small data set, which was an advantage brought about by a larger amount of data. The improved method had better accuracy in both large and small data sets, and could better extract and predict vulnerabilities compared to other models.

**Fig 14:**
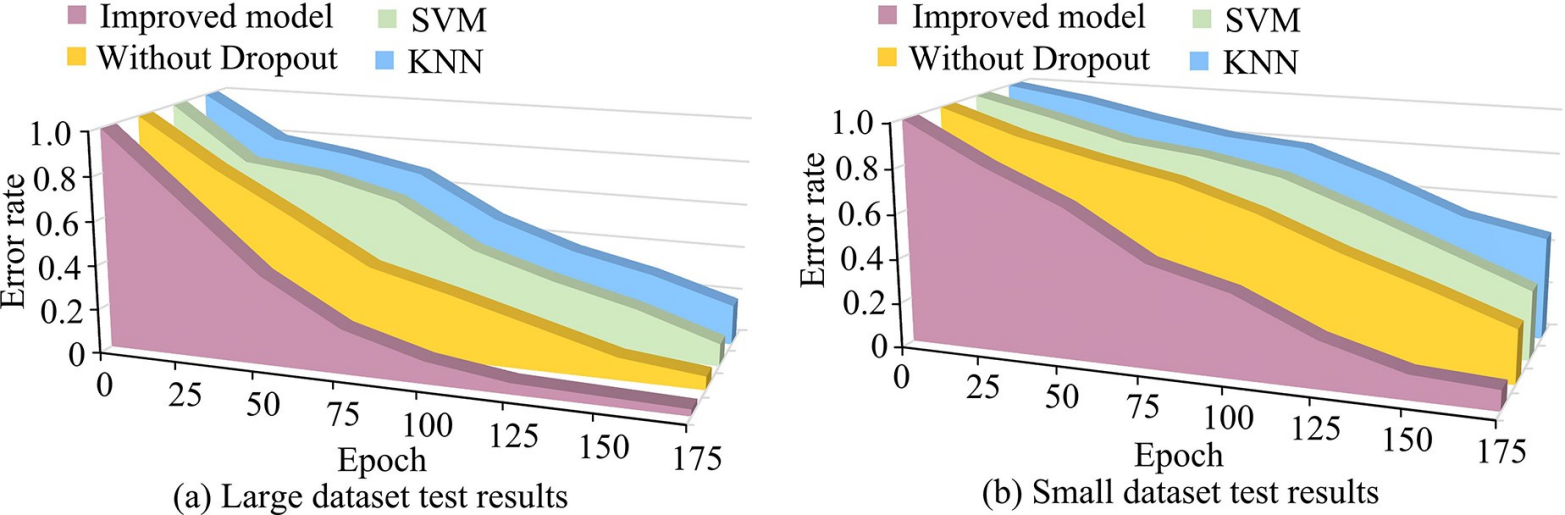
Comparison of error rates of four models.

The error rates of the four models were tested to evaluate their actual performance in vulnerability extraction and prediction. Run the model on the test set, collect the predictions, and then calculate the false positive rate of the model. The test results are shown in Fig 14. The overall error rate of big data set was significantly better than small data set’s, and the improved method had a minimum average error rate of 3.57%. It was 7.26% lower than the un-improved method, 14.67% lower than FF and 19.24% lower than KNN, respectively.

**Fig 15:**
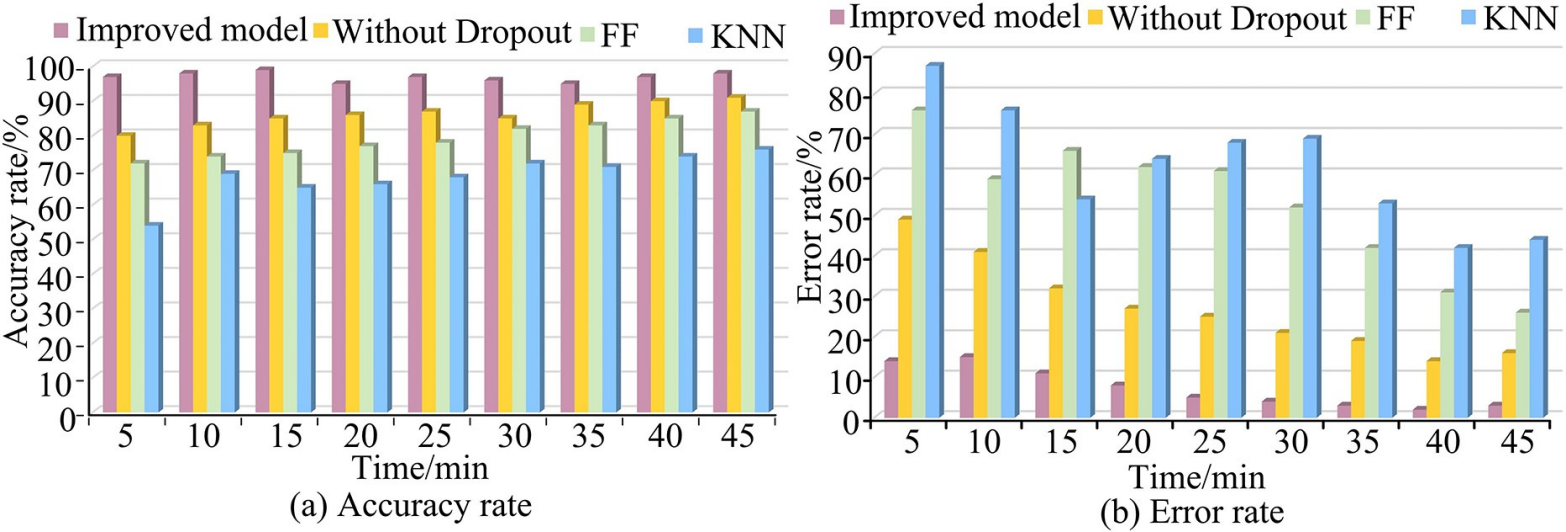
Accuracy & error rate of four models.

The Function fingerprints vulnerability detection method (FF) is further introduced [35]. In Fig 15, the actual testing was conducted for vulnerability detection in a simulated system. The proposed algorithm had higher vulnerability detection efficiency. As the running time increased, the vulnerability detection efficiency of other models significantly decreased, while the improved model could still maintain a high detection level. The impact of the running time on the improved model was relatively weak. Compared to other models, it could maintain higher accuracy over a long running time. And its error rate was less affected by running time and performed better.

**Fig 16:**
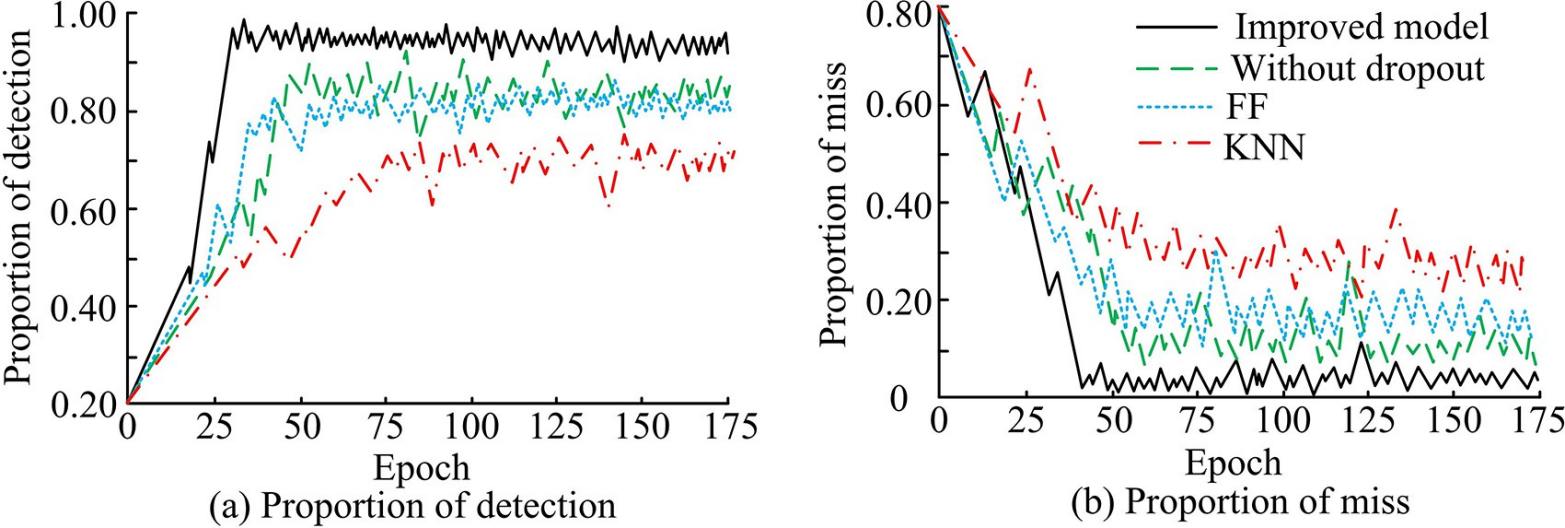
Proportion of detection & miss of four models.

Finally, in Fig 16, the actual vulnerability detection of the proposed model was tested using the method of actual testing comparison. The evaluation indicators were the proportion of vulnerabilities detected and the proportion of missed detections. The improved method performed well, with a vulnerability detection ratio of 95.37%, which was 8.11% higher than the unmodified model, 11.79% higher than FF and 23.48% higher than KNN, respectively. The final error detection ratio of its vulnerabilities was 4.62%, which decreased by 13.64% and 22.79% compared to FF and KNN, respectively.

In order to further verify the prediction accuracy of the model, the false positive rate and false negative rate of the four models were tested, and the test results were shown in Table 2. As can be seen from Table 2, the method proposed in this study has the lowest false positive rate and false negative rate. The overall false positive rate was 0.6% and the false negative rate was 0.3%, indicating that the proposed method has a high accuracy. In summary, the proposed vulnerability extraction and prediction method based on improved IG had shown good performance in testing. Moreover, it performed well in actual vulnerability extraction and prediction, and could effectively achieve vulnerability extraction and prediction, providing technical support for computer security.

**Table 2:** False positive rate and false negative rate test of four models.

Times	Models	Number of vulnerabilities	Detected count	False count	False positive rate	False negative rate
1	Improved model	20	20	2	1%	1%
	Without dropout		22	2	20%	8.3%
	FF		24	4	40%	20%
	KNN		26	5	45%	25%
2	Improved model	20	20	0	0%	0%
	Without dropout		21	2	15%	10%
	FF		23	3	35%	15%
	KNN		25	5	50%	25%
3	Improved model	20	20	2	1%	0%
	Without dropout		22	1	20%	5%
	FF		23	4	25%	20%
	KNN		25	6	40%	30%

The proposed method in this study and the latest model methods (including Coordinate system-based trust-aware model [31], Function fingerprints and code differences model [35] and Deep neural network-based botnet prediction model [20]) was compared, and the results were shown in Table 3.

**Table 3:** Performance comparison of the latest methods.

Method	Accuracy	Average forecast time	Reference
Coordinate system-based trust-aware	95.7%	653ms	Brahmi Z et al. [31]
Function fingerprints and code differences	93.9%	347ms	Zhao Q et al. [35]
Deep neural network-based botnet prediction model	95.1%	435ms	Haq M et al. [20]
Improved model	96.2%	332ms	This study

As can be seen from Table 3, the accuracy of Coordinate system-based trust-aware is 95.7%, and the average forecast time is 653ms. The accuracy of Function fingerprints and code differences was 93.9%, and the average forecast time was 347ms. The accuracy of Deep neural network-based botnet prediction model was 95.1%, and the average forecast time was 435ms. The accuracy of the Improved model was 96.2%, and the average forecast time was 332ms. It shows that the research method has better overall performance.

## 5. Conclusion

As the network develops, the significance of network security is more and more important. In the existing vulnerability database, due to the emergence of new data types and vulnerability types, the construction of vulnerability development chain cannot guarantee sufficient application period. Therefore, the study proposed a vulnerability extraction and prediction method based on improved IG, using DNN as the basic framework and Dropout to improve its IG ability, thereby improving its ability to utilize vulnerability feature data. These experiments confirmed that the F1 and Recall of this proposed method were excellent, with values of 0.972 and 0.968, respectively. Its F1 had been improved by 0.110 through Dropout, and its Recall had been improved by 0.107. The convergence performance of the improved method was excellent, and its performance was better than SVM and KNN. The response time of the proposed method was excellent, with a value of 0.12s, which was reduced by 0.16s and 0.21s compared to SVM and KNN, respectively. The proposed method had excellent predictive ability for vulnerability pre-permission and post-permission, with a pre-permission prediction accuracy of 97.9%, leading by 8.5% and 9.3% compared to SVM and KNN, respectively. Its post-permission prediction accuracy was 96.8%, leading by 5.1% and 11.6% compared to SVM and KNN, respectively. In the Mask testing, the proposed method performed much better than other methods, and it performed well even when faced with less data. SVM has strong high-dimensional data processing capability and excellent generalization performance, but SVM training requires a large amount of labeled data and has high computational costs. FF method is highly dependent on the existing vulnerability database, and its detection performance for new vulnerabilities or variants is limited. KNN, on the other hand, has relatively low data quality in feature selection and distance measurement, and poor performance in processing large-scale data sets. Compared with SVM and KNN, the research method has higher operational stability, excellent false positive rate and false negative rate, and higher vulnerability detection accuracy. Compared with the existing FF and KNN methods, the proposed method has stronger comprehensive performance, and also has the real-time and adaptability of vulnerability detection. This method can provide more accurate data support for the security system to help real-time monitoring and prevention of security vulnerabilities, especially in the dynamic network environment. By integrating into existing security architectures, it can enhance the ability to predict new and unknown vulnerabilities, providing enhanced data intelligence for security products such as firewalls and intrusion detection systems. This research can be widely cited in software development, cloud security, Internet of Things and critical network infrastructure protection, and improved vulnerability detection capabilities will greatly promote the development of network security. In the future, more advanced automation and accuracy will further improve the actual performance of research methods, and more current algorithms and models can be introduced to improve security vulnerability detection capabilities. However, the approach comes with its own set of limitations. The study did not expand the dataset, which means the breadth and depth of the vulnerabilities covered may be insufficient for the model to learn from and adapt to the full spectrum of real-world scenarios. Moreover, although the method is configured to perform in real-time, challenges persist when it comes to the resource demands of more complex algorithms, maintaining performance under high network loads, and ensuring the accurate prediction of unknown threats, such as zero-day vulnerabilities. Future studies should be carried out to improve its adaptability and complexity to enhance its performance. The generalization ability of the model can be enhanced by expanding the data set, the accuracy and robustness of the model can be improved by optimizing the combination of more advanced algorithms, and the efficiency of the model can be optimized to optimize its resource occupation.

## Supporting information

S1 File (DOC)
